# Impact of fractures and orthopedic surgeries in patients with HTLV-1 associated myelopathy/tropical spastic paraparesis

**DOI:** 10.1590/0037-8682-0101-2020

**Published:** 2020-09-11

**Authors:** Pablo Julca-Copello, Alvaro Schwalb, Rodrigo Cachay, Martín Tipismana, Carolina Alvarez, Fernando Mejía, Elsa González-Lagos, Eduardo Gotuzzo

**Affiliations:** 1 Universidad Peruana Cayetano Heredia, School of Medicine, Lima, Perú.; 2 Universidad Peruana Cayetano Heredia, Instituto de Medicina Tropical Alexander von Humboldt, Lima, Perú.; 3 Hospital Cayetano Heredia, Neurology Department, Lima, Perú.

**Keywords:** HTLV-1, HAM/TSP, Fractures

## Abstract

**INTRODUCTION:**

In patients with HTLV-1 associated myelopathy/tropical spastic paraparesis (HAM/TSP) gait disturbance is a predominant feature that leads to falls and fractures, which can further aggravate disability. We sought to evaluate the impact of fractures and orthopedic surgeries in patients with HAM/TSP.

**METHODS:**

We retrieved the medical records of HAM/TSP patients enrolled in our study center’s HTLV-1 clinical cohort between 1989-2018. The selection criteria included: (1) diagnosis of HTLV-1 infection using two enzyme-linked immunosorbent assays and/or a confirmatory test, (2) clinical diagnosis of HAM/TSP by neurological assessment, and (3) fractures associated with HAM/TSP.

**RESULTS:**

We identified 24 cases of fractures, 70% of which were females. The median age at the time of fracture was 60 years (IQR=24). Six cases reported fractures in patients under 45 years old. Ten patients (42%) had hip/coccyx fractures, seven (29%) were in the lower extremities, and four (17%) in the upper extremities. Half of these patients reported the use of wheelchairs. Five patients who had previously used canes required the use of wheelchairs after the reported fracture. Eight patients underwent corrective orthopedic surgery as a result of the fracture.

**CONCLUSIONS:**

For HAM/TSP patients, fractures are a complication that can exacerbate their severe impairment.

## INTRODUCTION

Human T-Cell Lymphotropic Virus Type 1 (HTLV-1) is a retrovirus associated with various clinical manifestations that affect approximately 10 million people worldwide^1^. The spectrum of diseases associated with HTLV-1 include adult T-cell leukemia/lymphoma (ATL), strongyloidiasis, crusted scabies, infective dermatitis, and inflammatory diseases such as uveitis and HTLV-1 associated myelopathy/tropical spastic paraparesis (HAM/TSP)^2^.

HAM/TSP, the most devastating neurologic consequence of HTLV-1, occurs in about 0.25-5% of infected individuals (usually adults in their fourth to fifth decade of life)^3^. The chronic progressive demyelination and degeneration of the first motor neurons determine the main clinical findings of spastic paraparesis, a positive Babinski reflex, and weakness in the lower extremities, among other signs of positive pyramidal release^4^ ^,^ ^5^. Consequently, many HAM/TSP patients present gait disturbances of varying frequency and severity. After 10 years of disease progression, approximately 50% of HAM/TSP patients require the use of a wheelchair, while others require crutches or canes to move^3^ ^,^ ^6^. A subgroup defined as rapid progressors (30% of cases) require a wheelchair earlier than 2 years from HAM/TSP onset^3^ ^,^ ^6^. As a result of gait abnormalities and lower extremity weakness, repeated falls are reported in two-thirds of HAM/TSP outpatients^7^. These falls may be forceful enough to generate non-spinal fractures, especially in the proximal femur^8^.

Given that little attention has been placed on fractures resulting from these falls, we aim to describe the impact of fractures in patients with HAM/TSP.

## METHODS

We identified a series of cases retrieved from the medical records of HAM/TSP patients participating in the HTLV-1 clinical cohort at the Instituto de Medicina Tropical Alexander von Humboldt (IMTAvH) in Lima, Peru, from 1989-2019. Routine evaluation of individuals with suspected and confirmed HTLV-1 infection has been provided at our institute. Evaluations include a standardized questionnaire by trained healthcare workers, thorough multi-disciplinary medical evaluation, and HTLV-1 testing for family members.

For this study, we selected cases reported within our medical records with (1) a diagnosis of HTLV-1 infection using two enzyme-linked immunosorbent assays and/or a confirmatory test (Western Blot), (2) clinical diagnosis of HAM/TSP by neurological assessment, and (3) a HAM/TSP-associated fracture, defined as a fracture occurring after HAM/TSP diagnosis and not associated with major trauma, such as automobile accidents.

Data retrieved included: sex, age at time of fracture, fracture location, need for corrective orthopedic surgery due to fracture, type of mobility aids required by the patient (canes, wheelchairs, etc.), and associated symptoms including a tendency for falls, gait disturbances, and lower back pain. The presence of osteoporosis by bone densitometry was also noted, although this test was not routinely performed in the HTLV-1 clinical cohort. During different appointments in the patient’s follow-up, HAM/TSP severity was measured subjectively, and the Kurtzke Expanded Disability Status Scale (EDSS) was applied to measure the patient’s functional capacity^9^. The EDSS evaluates 8 functional systems including pyramidal, cerebellar, brainstem, sensory, bladder/bowel, visual, mental, and other. 

Patient information is stored in a Microsoft Access database designed for the HTLV-1 cohort. The data are expressed as frequencies for categorical variables and as the mean and standard deviation or median and interquartile range (IQR) for continuous variables, depending on the distribution. All the analyses were done using Stata SE 15 (StataCorp., US, license Universidad Peruana Cayetano Heredia). The study protocol was approved by the Institutional Ethics Committee of Universidad Peruana Cayetano Heredia (SIDISI: 103668)*.*

## RESULTS

Since the first reported fracture in 2007, 719 new patients with HAM/TSP have been included in our clinical cohort. We identified 23 patients with fractures associated with HAM/TSP, one of them presenting two distinct fractures. Of those 23 patients, 70% were female. The median age at the time of fracture was 60 (IQR, 24; range, 30-79) years; notably, six of these cases were in patients younger than 45 years old. Fractures were mostly reported in the hip/coccyx (n = 10; 42%) and in the lower extremities (n = 7; 29%) (Table 1). We found five cases of HAM/TSP that were classified as severe (21%) after the fracture episode.

According to the EDDS, six patients scored between 4.5-7 during follow-up after the fracture episode. We also found that, at the time of the fracture, two patients already had scores of 6 and 6.5 on the scale.

**TABLE 1:** Characteristics of patients with fractures associated with HAM/TSP.

Characteristics	n = 24	
	n	%
Sex (n = 23)		
Female	16	70
Male	7	30
Age of fracture (years)	60*	
Place of fracture		
Hip/coccyx	10	42
Lower extremities	7	29
Upper extremities	4	17
Others	3	13
Corrective orthopedic surgery (n = 23)	8	35
Gait problems (n = 23)	22	96
Lower back pain (n = 16)	14	88
Falls (n = 20)	14	70

*Median, interquartile range: 45-69. **HAM/TSP:** HTLV-1 associated myelopathy/tropical spastic paraparesis.

As a result of fracture, eight patients (35%) required a corrective surgical procedure and five required hip surgery (Table 2). Only three patients had bone densitometry testing performed after the fracture episodes, which were indicative of osteoporosis. 

**TABLE 2:** Fracture characteristics and management among patients with HAM/TSP.

N°	Sex	Age of fracture (years)	Location of the fracture	Corrective surgical procedure	Mobility aid prior to fracture	Mobility aid after fracture
1	F	75	Hip	Yes	-	-
2	M	34	Hip	Yes	Wheelchair	Wheelchair
3	F	45	Ankle	Yes	-	Wheelchair
4	F	63	Foot	No	Cane	Wheelchair
5	F	68	Hip	No	-	Wheelchair
6	F	30	Hip	-	-	Wheelchair
7	M	40	Elbow	No	-	-
8	F	79	Hip	Yes	-	-
9	F	-	Coccyx	No	Wheelchair	Wheelchair
10	F	41	Hip	Yes	-	Wheelchair
11	M	67	Femur	No	-	Cane
	M	75	Hip	Yes	Cane	Cane
12	M	58	Hip	No	Cane	Wheelchair
13	M	-	Nasal septum	No	-	-
14	M	71	Vertebrae D4 and L1	No	Cane	Cane
15	F	-	Multiple fractures	No	-	Wheelchair
16	F	56	Humerus	No	Cane	Cane
17	F	52	Tibia and fibula	Yes	Cane	Wheelchair
18	F	63	Tibia	No	Cane	Wheelchair
19	F	78	Tibia and fibula	No	Cane	Wheelchair
20	M	60	Arm	Yes	-	Cane
21	F	60	Foot	No	-	Cane
22	F	41	Humerus	No	Cane	-
23	F	69	Coccyx	No	-	-

**HAM/TSP:** HTLV-1 associated myelopathy/tropical spastic paraparesis. **F:** Female; **M:** Male.

Clinically, 96% of patients presented gait disturbances, 88% reported lower back pain, and 70% had had episodes of falls during the course of the disease and follow-up (Table 1). Nine patients (38%) used canes as mobility aids; however, after fracture, five of them required the use of wheelchairs. Half (52%) of the patients reported the use of wheelchairs (Table 2).

## DISCUSSION

In this study, we identified 24 fractures associated with HAM/TSP. Although a small percentage (3.2%) of fractures had been reported among our HAM/TSP patients, these accidents impact the patient's quality of life by increasing the need for mobility aids and healthcare-related expenses. The prevalence of falls ranges between 39-49% among those aged >60 years old^10^, and it has been reported in two-thirds of HAM/TSP patients^7^. These findings reinforce the idea that HAM/TSP patients have an increased risk of falls and, consequently, of fractures^11^ ^,^ ^12^. Gait impairment is a major risk factor associated with falls; the reduction in the functional angle of the hip, knee, and ankle leads to compensatory axial movements and, ultimately, the need for mobility aids^13^.

In a previous study by Gotuzzo et al., it was observed that two-thirds of patients with HAM/TSP from the HTLV-1 clinical cohort at the IMTAvH (up to 2002) were unable to walk without aid^14^ ^,^ ^15^. Our series reports two-thirds of patients as wheelchair users after fracture. We found that in five patients the type of mobility aid changed from canes to wheelchairs after fracture, indicating a greater reliance on mobility aids. Moreover, Caiafa et al. links wheelchair use to muscular weakness in HAM/TSP patients, further increasing dependency^16^.

Distal paresthesia and weakness of the lower extremities in HAM/TSP predisposes individuals to falls^17^. Falls account for the majority of hip fractures in elderly patients^18^; likewise, this was the most common place for fractures followed by lower extremities among our patients. It is important to highlight that six out of 23 patients that suffered fractures were younger than 45 years of age, causing impairment at a young age. Osteoporosis was diagnosed in three female patients by bone densitometry (DEXA) scan; nonetheless, this might be an underestimation as not all patients in the cohort were routinely tested. According to one study, HAM/TSP patients present lower T-scores, some even below -2.5, which defines osteoporosis^19^. Another study shows that the low T-scores are the result of high serum levels of parathyroid hormone found in HTLV-1 infection^20^.

Most fractures were in the hip/coccyx, five fractures required corrective surgery, which generates an important bidirectional impact between the patient's quality of life and costs surrounding the fracture, both to the patient and healthcare system. The estimated incidence of hip fractures is greater among females than in males (444 vs 264 per 100,000 inhabitants, respectively)^21^. According to the fare listing from a public hospital in Peru in 2017, the cost for a hip hemiarthroplasty is 302 PEN (91 USD) for a patient with health insurance and 616 PEN (187 USD) for a patient without insurance. The pre-op median length of stay for a hip fracture is 18 days, however, patients may remain in the hospital for 1-3 months after the surgery^22^. Considering surgical materials, prosthetic implants, anesthetics, and length of stay at the hospital, the total cost would be approximately 13,000 PEN (3,940 USD) in a public hospital. In a private clinic, this amount may be three-fold higher. Costs may slightly reduce for patients enrolled in the global health insurance program (SIS) only if the hospital is properly stocked with the materials for the surgery.

The main limitation of our study was that fractures were self-reported and not confirmed through access to the hospital X-ray records. Nonetheless, we believe this may not have led to an overestimation of the number of fractures. Additionally, the use of mobility aids prior to a fracture is not explicitly reported in the patient charts which could mean that their gait assistance was minimal or occasionally result in an underreporting of mobility aids. Furthermore, bone density tests were not routinely performed limiting the assessment of the contributing role of osteoporosis among our patients.

Our study found that fractures and subsequent orthopedic surgeries are an unexplored consequence in patients with HAM/TSP. These complications inflict great impairment in the lives of these patients, mostly by placing them in a wheelchair. Associated expenses are also a burden to the patient and to the healthcare system; these could potentially be decreased with proper rehabilitation when gait disturbances appear. These cases should help highlight the need for larger studies to understand the true impact of this disabling condition.
